# Development and Characterization of Cannabidiol Self-Emulsifying Drug Delivery System: In Vitro and In Vivo Evaluation

**DOI:** 10.3390/biom16010021

**Published:** 2025-12-23

**Authors:** Nourhan Mostafa, Iman E. Taha, Noha M. Abourobe, Eman A. Ashour

**Affiliations:** 1 Department of Pharmaceutics and Drug Delivery, School of Pharmacy, University of Mississippi, University, MS 38677, USA; nsmostaf@go.olemiss.edu (N.M.);; 2 Department of Pharmaceutics, Faculty of Pharmacy, Suez Canal University, Ismailia 41522, Egypt; noha_ibrahim@pharm.suez.edu.eg

**Keywords:** cannabidiol (CBD), endocannabinoid system (ECS), lipid-based drug delivery system (LBDDS), self-emulsifying drug delivery system (SEDDS), non-ionic surfactant, inhibition effect, bioavailability

## Abstract

Cannabidiol (CBD) is a non-psychoactive phyto-cannabinoid with numerous pharmacological potentials. CBD is a lipophilic drug with poor and varied bioavailability due to its low water solubility and extensive first-pass metabolism, and it is highly affected by the presence of food. A self-emulsifying drug delivery system (SEDDS) was developed to improve the aqueous solubility and oral bioavailability of CBD. The formulation strategy involved incorporating excipients that maintain drug solubility under both fasted and fed conditions, while potentially mitigating first-pass metabolism to enhance overall bioavailability and dose proportionality. Caproyl^®^ 90, Tween^®^ 20, and Transcutol^®^ HP were selected as the oil phase, surfactant, and cosolvent, respectively, for formulation preparation and screening. CBD SEDDS formulations containing Caproyl^®^ 90 ≤20% *w*/*w* and Tween^®^ 20 above 40% *w*/*w* yield particles below 200 nm. CBD SEDDS with Tween^®^ 20 65% *w*/*w* or higher showed in vitro release of more than 90%. After in vitro digestion, CTT1, CTT4, and CTT8 remained stable under gastrointestinal conditions and maintained CBD solubility of at least 50%. The most promising formulations, CTT4 and CTT8, were used for in vivo evaluations. Both formulations showed similar in vitro results; however, in vivo, CTT4 demonstrated 2.4-fold higher bioavailability than CTT8. Overall, optimizing the level of inhibitory surfactant appears to be a promising strategy for improving CBD bioavailability.

## 1. Introduction

*Cannabis sativa* L. is one of the oldest known medicinal plants [1]. Systematic scientific study began in the 19th century with O’Shaughnessy, who documented its pharmacological effects [2,3]. The first cannabinoid, cannabinol, was isolated in the late 1800s, followed by cannabidiol (CBD) in 1940 and the primary psychoactive compound Δ9-tetrahydrocannabinol (THC) in 1964 [4,5,6,7]. Subsequent research led to the identification of cannabinoid receptors (CB1 in the late 1980s) and endogenous cannabinoids, establishing the framework of the endocannabinoid system [7,8,9]. The endocannabinoid system (ECS) is a neuromodulatory network widely distributed in the brain [10]. CB1 receptors are abundant in the central and peripheral nervous systems and are involved in regulating pain, mood, and emotional processing. CB2 receptors, originally linked mainly to immune function, are also present in the brain and may influence neural activity [11,12].

Cannabidiol (CBD) is a non-psychoactive phyto-cannabinoid with numerous beneficial pharmacological effects, including anti-inflammatory, antioxidant, anxiolytic, antidepressant, antipsychotic, and anticonvulsant properties [13,14]. The molecular mechanism of CBD and its therapeutic benefits are suggested to be mainly generated from CBD’s interaction with the ECS [15]. CBD has a very low affinity for both cannabinoid receptors compared to THC, and that explains why CBD is non-psychoactive. Furthermore, CBD decreases CB1 receptor activation, which can inhibit the psychoactive nature of THC by exhibiting behavior similar to an antagonist (negative allosteric modulation) [16]. However, CBD can indirectly target CB1 receptors by inhibiting the fatty acid amide hydrolase (FAAH) enzyme, which degrades the endogenous cannabinoid anandamide, leading to an increase in anandamide concentration at synapses [17].

Epidiolex^®^, a CBD oral solution, was FDA-approved after demonstrating its positive effects on a wide range of seizures. Preclinical evidence suggests that CBD has anticonvulsive properties [18]. CBD had a significant effect on children and adults with Lennox–Gastaut syndrome, when administered a dose of either 10 mg or 20 mg/kg/day, it led to at least a 50% reduction in the frequency of drop seizures, the decrease in the frequency of all seizures, with an improvement in overall condition, and 5% of patients were free from drop seizers and these results similar to trial CBD at dose of 20 mg/kg/day in patients with the Dravet syndrome [19].

From a physico-chemical perspective, CBD is poorly water soluble (12.6 mg/L) and has a high lipophilicity (log P of 6.3) [20]. CBD undergoes extensive first-pass metabolism, resulting in a low absolute oral bioavailability of approximately 6%. Furthermore, CBD bioavailability is highly food-dependent, increasing fourfold when co-administered with a high-fat meal, leading to high inter- and intra-subject variability and a lack of dose proportionality [20,21]. Epidiolex^®^ bioavailability can vary from 4.36% to 57.30% depending on dose and fasting or fed state. In the fasted state, the dose influences bioavailability, exhibiting a nonmonotonic, inverted-U-shaped profile. For a low dose of CBD (50 mg), oral bioavailability is estimated to be 4.36%, as most of the drug undergoes first-pass metabolism. Increasing the dose increases bioavailability until reaching the GIT fluid solubility limit at doses of 750 mg or more, after which bioavailability decreases [22].

The fact that the oral absorption of poorly water-soluble drugs and their bioavailability can be improved when administered with a high-fat meal has led to the use of lipid-based drug delivery (LBDD) formulations as a strategy to enhance drug solubility and absorption after oral administration by creating an effect similar to the presence of food [23,24]. LBDDS can increase drug absorption from the gastrointestinal tract (GIT) by maintaining the drug in a solubilized state in GIT fluids. Furthermore, LBDDS may enhance oral bioavailability by improving the multiple mechanical pathways, such as extending the gastric residence time, facilitating intestinal to lymphatic transport (thereby avoiding first-pass metabolism), enhancing intestinal epithelium permeability and allowing paracellular transport by opening tight junctions and reducing efflux transporters’ activity by inhibiting P-glycoprotein (P-gp), and mitigating metabolic property by inhibiting CYP450 enzymes [25,26].

A self-emulsifying drug delivery system (SEDDS) is a type of LBDDS. SEDDS consist of an isotropic mixture of drug, oils, surfactants, and co-surfactants/co-solvents. It is generally described as an emulsion pre-concentrate upon mild agitation, followed by dilution in aqueous media, such as GIT fluids, which would form an O/W emulsion [27,28]. SEDDS has advantages over other traditional emulsions, which require high shear to generate a dispersion. SEDDS preparation is easy to manufacture and scale up, as the drug is simply dissolved in oil and then combined with surfactants, and co-surfactants/cosolvents. SEDDS formulations are more physically and chemically stable because they are isotropic mixtures, do not contain any water, and do not undergo creaming over storage [29,30,31].

Oil is essential for drug solubilization. Long-chain glycerides are essential for the transport of drugs through lymphatic vessels. On the other hand, medium-chain glycerides are absorbed through the portal veins, and they are the most commonly used oils, as they improve the quality of solubility and oxidation resistance [30,31]. Surfactants are essential for reducing the surface tension between the oil and aqueous phase and facilitating the dispersion process. In addition, surfactants impact the efflux transporter, intra-enterocyte metabolism, and intestinal permeability [32]. Using a high concentration of a single surfactant is rarely able to provide low interfacial tension; therefore, the addition of another surfactant (cosurfactant) or cosolvent is usually necessary. They can synergistically cooperate to enhance the solubility of the drug and the dispersibility of the surfactant in the oil, thereby promoting the stability and homogeneity of the emulsion [33].

The objective of this study is to enhance CBD bioavailability by designing stable CBD SEDDS formulations that can decrease CBD hepatic metabolism by utilizing medium-chain oil to maximize drug exposure to portal circulation, as well as by incorporating a surfactant that has shown inhibitory activity against CYP 450. This study will examine these effects on the in vivo bioavailability.

## 2. Materials and Methods

### 2.1. Material

Cannabidiol (CBD) was received as a gift from ElSohly Laboratories, Inc. (eLi) (Oxford, MS, USA). Propylene Glycol Monocaprylate NF (Capryol^®^ 90), Caprylocaproyl Polyoxyl-8 glycerides (Labrasol^®^), Oleoyl polyoxyl-6 glycerides (Labrafil^®^ M 1944 CS), Polyglyceryl-3 dioleate NF (Plurol^®^ Oleique CC 497), and highly purified diethylene glycol monoethyl ether EP/NF (Transcutol^®^ HP) were gifted by Gattefossé (Paramus, NJ, USA). Polyoxyethylene sorbitan monolaurate (Tween^®^ 20), polyoxyethylene sorbitan monooleate (Tween^®^ 80), and Sesame oil were obtained from Fisher Scientific (Hanover Park, IL, USA). Polyethoxylated castor oil (Kolliphor^®^ EL) and Porcine pancreatin extract (8 ×USP specifications activity) were purchased from Sigma-Aldrich (Louis, MO, USA). Biorelevant 3F Powder™ was obtained from Biorelevant (London, UK). All solvents used were HPLC grade.

### 2.2. Methods

#### 2.2.1. HPLC Analytical Method

CBD was quantified using a Waters HPLC-UV system (Waters Corporation, Milford, MA, USA) and a Phenomenex Luna column [5 µm C18 (2) 100 Å, LC, 150 × 4.6 mm]. The mobile phase consisted of 23% (*v*/*v*) water and 77% (*v*/*v*) acetonitrile with 0.1% (*v*/*v*) formic acid, at a flow rate of 1 mL/min. The detection wavelength was 220 nm [34]. Calibration curves were constructed with concentrations ranging from 10 to 250 µg/mL.

#### 2.2.2. Screening of CBD Solubility in Different Excipients

The solubility of CBD was evaluated in various excipients (Tween^®^ 20, Tween^®^ 80, Kolliphor^®^ EL, Labrasol^®^, Transcutol^®^ HP, Plurol^®^ Oleique CC 497, Labrafil^®^ M 1944 CS, sesame seed oil, and Capryol^®^ 90) to determine the oil, surfactant, and cosurfactant/cosolvent that provides the highest CBD solubility. An excess amount of CBD (200 mg) was added to 1 mL of each excipient, followed by vortex mixing for 30 s to ensure uniform dispersion and facilitate solubilization of the CBD within the excipient. The samples were then kept on a rotary shaker (VWR, Radnor, PA, USA) at room temperature at 100 rpm for 72 h to allow the system to reach equilibrium [35]. Subsequently, the mixtures were centrifuged at 13,000 rpm for 15 min to separate undissolved CBD. The supernatant was carefully collected and diluted 2000-fold with acetonitrile. The CBD concentration was then quantified using a validated HPLC method, as described in Section 2.2.1.

#### 2.2.3. Pseudo-Ternary Diagram Construction

Based on the solubility study of CBD in different excipients and preliminary screening, a pseudo-ternary diagram was constructed using Capryol^®^ 90 as an oil phase, Tween^®^ 20 as the surfactant, and Transcutol^®^ HP as a cosolvent. For any formulation, the concentration of each excipient varied from 10% *w*/*w* and 80% *w*/*w*, maintaining a total mass percentage of 100% *w*/*w* for each mixture. Sixteen formulations (Table 1) were prepared and kept at room temperature for 48 h to assess their physical stability and observe any signs of phase separation. The corresponding zone in the diagram was used to determine the specific range of excipient concentrations capable of forming dispersible systems under dilution with gentle agitation. Following dispersion, the emulsions were visually evaluated to characterize their appearance, such as transparent emulsion, milky, or oily phase separation. Formulations that demonstrated acceptable dispersion behavior were further characterized for particle size (PS), polydispersity index (PDI), and emulsification time (ET).

#### 2.2.4. Emulsification Time

A total of 0.5 mL of the liquid SEDDS formulation was added to 20 mL of Milli-Q water, and the mixture was gently stirred at 100 rpm and 37 °C. The emulsification process was visually monitored until complete dispersion of the formulation was achieved, and ET was recorded.

#### 2.2.5. Particle Size and Polydispersity Analysis

After complete dispersion of the SEDDS formulations, the emulsion was diluted 10-fold with Milli-Q water. The PS and PDI were then assessed using a Zetasizer instrument (Zen3600, Malvern Panalytical Inc., Westborough, MA, USA) at a scattering angle of 90° and a temperature of 25 °C [36].

#### 2.2.6. Drug Loading

Based on the pseudo-ternary phase diagram and the PS and PDI data of the placebo formulations, the optimal compositions were selected for drug loading. CBD (1 g) was incorporated into 10 g of the selected placebo formulations and stirred using a magnetic stirrer at 600 rpm and 50 °C for 30 min until complete dissolving was achieved. The CBD-loaded formulations were then evaluated for drug content, ET, PS, and PDI.

#### 2.2.7. Thermodynamic Stress Testing for Kinetic Stability Evaluation

The thermodynamic stress testing for the CBD SEDDS formulations was evaluated to determine the effects of stress conditions and temperature fluctuations on the physical stability of the SEDDS formulations. The evaluation involved centrifugation and freeze–thaw cycle tests. During centrifugation, the CBD SEDDS formulations were centrifuged at 6000 rpm for 15 min to detect any phase separation; only stable formulations that showed no signs of separation were selected for further testing. On the other hand, a freeze–thaw cycle was conducted through three cycles of freeze–thaw, which were executed at −20 °C, room temperature, and at 40 °C. At each temperature, the formulations were maintained for a minimum of 48 h [37]. Following the completion of the stress test, the CBD SEDDS formulations were analyzed for their ET, PS, and PDI to confirm their structural integrity.

#### 2.2.8. In Vitro Release

The in vitro drug release profile of CBD from the CBD SEDDS formulations was evaluated and compared with that of pure CBD using the USP Dissolution Apparatus II (paddle). The test was conducted in 900 mL of phosphate buffer (pH 6.5) maintained at 37 °C ± 0.5 °C with a paddle rotation speed of 50 rpm. About 1 g of CBD SEDDS formulation (equivalent to 90.09 mg of CBD) was introduced to the dissolution medium. 1.5 mL samples were withdrawn at predetermined time points (5, 15, 30, 45, 60, 90, and 120) minutes. The samples were then centrifuged at 13,000 rpm for 15 min, and the supernatants were collected and quantitatively analyzed for CBD using HPLC.

#### 2.2.9. In Vitro Digestion

The in vitro digestion studies were performed on selected CBD SEDDS formulations (CTT1, CTT4, and CTT8) that showed in vitro release of >90%. The study protocol was adapted from previously published methods [38,39]. Simulated intestinal media representing fasted and fed physiological states were prepared using Biorelevant™ media components. For fasted state simulated intestinal fluid (FaSSIF) digestion media, malic acid buffer (pH 6.5) was prepared by dissolving maleic acid (C_4_H_4_O_4_, 19.12 mM), sodium hydroxide pellets (NaOH, 34.80 mM), sodium chloride (NaCl, 68.62 mM), and calcium chloride dihydrate (CaCl_2_ · 2H_2_O, 1.40 mM). The Biorelevant 3F Powder™ was reconstituted in the malic acid buffer according to Biorelevant manufacturing instructions. For the fed state simulated intestinal fluid (FeSSIF), calcium chloride dihydrate (1.4 mM) was added to the Biorelevant acetate buffer (pH 5.0) and combined with Biorelevant 3F Powder™ as per the provided preparation guidelines. Lipolytic enzymes were prepared by mixing 1 g of porcine pancreatin powder in 5 mL of digestion buffer (without Biorelevant 3F Powder™). The suspension was centrifuged at 4000 rpm for 15 min, and the supernatant containing the enzyme fraction was collected for use.

For each in vitro digestion experiment, 1 g of CBD SEDDS formulation (in triplicate) was directly dispersed into 36 mL of preheated digestion buffer (37 °C) under continuous stirring at 100 rpm for 10 min. After dispersion, a 1 mL sample was withdrawn and replaced with fresh medium to maintain constant volume. Digestion was initiated by adding 4 mL of pancreatin extract. During the digestion, the pH was continuously monitored and maintained at 6.5 for FaSSIF and 5.0 for FeSSIF using 0.6 M NaOH. The digestion process was continued for 30 min. At the end of the digestion test, 1 mL samples were collected, immediately filtered through a 0.45 μm polyvinylidene fluoride (PVDF) syringe filter to remove undissolved CBD. The samples were then diluted with a chloroform/methanol mixture (1:2, *v*/*v*). The concentration of solubilized CBD was subsequently quantified using HPLC.

#### 2.2.10. In Vivo Bioavailability Study

In vivo bioavailability studies were conducted on the formulations that exhibited the highest CBD solubility in the digestive media, using a method adapted from our previously published study [40]. According to approved procedures and protocols by the Institutional Animal Care and Use Committee (IACUC) of the University of Mississippi under protocol number 22−003, approval date is 19 January 2022. The study was conducted using Sprague-Dawley rats (200–250 g) divided into three groups, each consisting of three rats. The rats were catheterized with a jugular catheter. One group received a CBD solution administered intravenously (CBD IV) containing 1 mg of CBD in a mixture of Cremophor, ethanol, and water (1:1:8), and the other two groups received one of the CBD SEDDS formulations, CTT4 or CTT8, containing 5 mg of CBD by oral gavage. All the groups were fasted overnight and housed separately in a controlled laboratory environment. Post-administration, (200–300 µL) of blood samples were withdrawn at 0, 5, 10, 15, and 30 min, and at 1, 2, 4, 6, and 24 h. The blood samples were centrifuged at 3000 rpm for 5 min at 4 °C to collect the plasma, which was then stored at −80 °C until analysis.

##### Plasma Samples Preparation

A simple protein precipitation method was used for CBD extraction from the rat’s plasma by taking 50 µL of each plasma sample and placing it into a 0.5 mL Eppendorf tube, followed by treating the samples with 100 µL of 100% acetonitrile with an internal standard (20 ng/mL of CBD-d3 and tolbutamide) and then mixing for 15 s on a cyclomixer (Thermo Scientific, Indianapolis, IN, USA). After that, samples were vortexed for 2 min and centrifuged for 15 min at 14,000 rpm on an AccuSpin Micro 17R (Fisher Scientific, Suwanee, GA, USA) at 4 °C. The clear supernatant (approximately 80 µL) was transferred to LC-MS/MS vials for analysis.

##### Plasma Sample Analysis

For plasma sample analysis, an ultra-performance liquid chromatography (UPLC) system (Waters XevoTM TQ-S Acquity UPLCTM, Waters Corp., Milford, MA, USA) with an autosampler temperature of 10 °C and a column oven temperature of 40 °C was used. For chromatographic separation, A Waters Acquity UPLC^®^ BEH Phenyl column (50 mm × 3.0 mm × 1.7 μm) was used with a linear gradient elution consisting of 90% acetonitrile and 10% 0.2% formic acid in Milli-Q water as mobile phases. The running time was 5.0 min, with a flow rate of 0.3 mL/min, and an injection volume of 2 µL. A mixture of 80% methanol and 20% Milli-Q water was used for rinsing. For sample detection, an Acquity Tandem Quadrupole Mass Detector (Xevo TQ-S; Waters Corp, Milford, MA, USA) in positive electro-spray ionization mode was used for CBD detection. For quantification, multiple reaction monitoring (MRM) was used. The CBD calibration curve ranged from 5.6 to 560 ng/mL, while the Lowest Limit of Quantification (LLOQ) was 5.6 ng/mL. The oral bioavailability of the SEDDS formulations was calculated from the area under the curve (AUC) using the following equation:

$$Oral\;Bioavailability\;\left(\%\right)=\;\frac{AUC\;\left(oral\right)\times \;Dose\;(IV)}{AUC\;\left(IV\right)\times \;Dose\;(oral)}\;\times \;100$$

## 3. Results and Discussion

### 3.1. Solubility Study

Selecting suitable excipients is a critical step in developing SEDDS formulations, as they determine the system’s solubilizing capacity and directly influence the achievable drug loading. The mixture of oil, surfactant, cosolvent, and drug should be physically stable, form a clear, homogeneous system to ensure uniform drug distribution throughout the formulation, and preserve its self-emulsifying characteristics. [28]. Moreover, MCTs are absorbed via the portal veins, providing a clear in vivo image of the effect of surfactant CYP inhibition on CBD bioavailability. Capryol^®^ 90 is a medium-chain monoglyceride that consists of 8 carbons [41]. For surfactant selection, it is essential to consider that CBD is primarily metabolized in the liver by cytochrome P450 enzymes CYP3A4 and CYP2C19, which are the major hepatic CYP3A and CYP2C enzymes, respectively [42]. Therefore, designing CBD SEDDS formulations with non-ionic surfactants such as polyoxyethylene sorbitan fatty acid esters and polyoxyethoxylated castor oil, which are capable of inhibiting CYP3A and CYP2C [43], may help reduce hepatic metabolism and enhance bioavailability.

**Figure 1:**
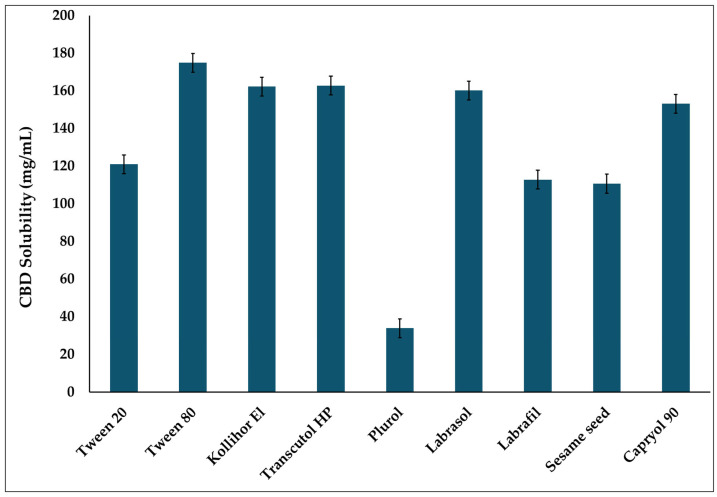
Solubility of CBD in different excipients (Mean ± SD, *n* = 3).

According to the literature, a study by Anne Christiansen et al. found that the non-ionic surfactant inhibited CYP2C9 and CYP3A4 in vitro in a concentration-dependent manner. Among the surfactants they screened, Tween^®^ 80 moderately inhibited both enzymes. In contrast, they found Kolliphor^®^ EL more effective at inhibiting CYP2C19 than at inhibiting CYP3A4 [44]. In another study by Xiuhua Ren et al., multiple non-ionic surfactants were examined, and it was concluded that Tween^®^ 20 is the most potent CYP3A inhibitor. And for in vivo evaluation, they reported that after a single dose, the AUC_0–4h_ of midazolam increased 140%, while its metabolites decreased to 36% [45]. Therefore, in this study, Kolliphor^®^ EL, Tween^®^ 80, and Tween^®^ 20 were screened to select the most suitable surfactant for CBD SEDDS formulations (Figure 1). CBD showed high solubility in Tween^®^ 80, Kolliphor^®^ EL, and Tween^®^ 20. However, during the solubility assessment, an unexpected color change was observed in the vial containing Kolliphor^®^ EL, which turned red. This observation suggested a potential chemical instability between Kolliphor^®^ EL and CBD; therefore, Kolliphor^®^ EL was excluded from further consideration. To compare Tween^®^ 80 and Tween^®^ 20 as surfactants, SEDDS formulations were prepared using a precise formulation composition consisting of 20% *w*/*w* oil (Capryol^®^ 90), 65% *w*/*w* surfactant (Tween^®^ 80 or Tween^®^ 20), and 15% *w*/*w* cosolvent (Transcutol^®^ HP). The SEDDS formulation with Tween 80 exhibited a particle size (117.40 ± 35.11 nm), with a polydispersity index (PDI) of (0.40 ± 0.27) with a relatively high standard deviation for the particle size, and a high PDI (above 0.3), indicating non-uniform dispersion, which could negatively affect the physicochemical characteristics and, subsequently, drug absorption [46,47]. In contrast, the formulation prepared with Tween^®^ 20 showed a consistent particle size (13.39 ± 1.033 nm) and PDI (0.294 ± 0.061). The smaller PS and lower PDI observed in the emulsions prepared with Tween^®^ 20 may be attributed to its higher hydrophilic–lipophilic balance (HLB) (HLB 16.7) than that of Tween^®^ 80 (HLB 15). The higher HLB of Tween^®^ 20 results in greater accumulation in the hydrophilic environment, leading to smaller, more uniform, and more stable dispersed droplets [48]. These particle sizes and PDI values make Tween^®^ 20 the preferred surfactant.

### 3.2. Pseudo-Ternary Diagram

After selecting the oil, surfactant, and cosolvent for the SEDDS formulations based on the results of the solubility study, a pseudo-ternary diagram was constructed. This diagram is crucial for understanding how different ratios among these components can affect the system performance and determining the probable proportions of the excipients required to develop a fine emulsion in the nano range. The combination of Capryol^®^ 90, Tween^®^ 20, and Transcutol^®^ HP demonstrates high compatibility and stability over 48 h, as indicated by the absence of phase separation in all formulations. After confirming the physical stability of placebo SEDDS formulations, their emulsification behavior was evaluated. According to the pseudo-ternary diagram, formulations with high oil content (40% *w*/*w* or higher) tend to exhibit partial phase separation after emulsification, forming oil globules on the emulsion surface. In contrast, maintaining the oil concentration below 20% yielded transparent emulsions with droplet sizes below 65 nm, whereas formulations with oil concentrations between (20–40%) yielded a turbid emulsion with larger particle sizes.

**Figure 2:**
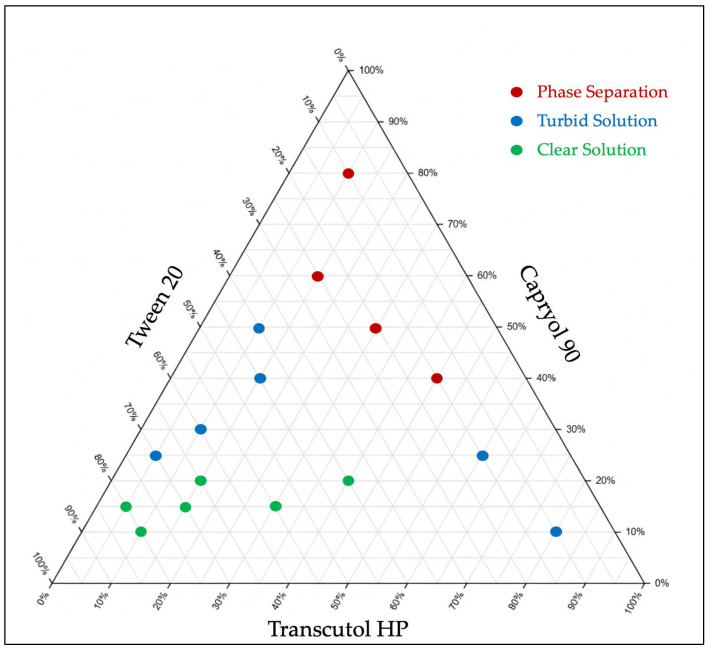
Pseudo-ternary diagram of SEDDS formulations without drug, using Capryol^®^ 90 as the oil, Tween^®^ 20 as the surfactant, and Transcutol^®^ HP as the cosolvent.

On the other hand, there was an inverse relationship between the concentrations of surfactant and cosolvent and the PS. Increasing the concentrations of the surfactant and cosolvent would help decrease the emulsion PS (Table 2). The optimum PS was achieved by increasing the Tween^®^ 20 portions above 40% *w*/*w* and keeping the Transcutol^®^ HP concentration between 5 and 40% *w*/*w* (Figure 2).

### 3.3. Emulsification Time

ET refers to the duration required for the formulation under gentle agitation to produce a homogeneous emulsion. Short emulsification time could contribute to a quick drug release and a rapid onset of action. In some cases, emulsification time may be dependent on the concentration of the oil-to-surfactant ratio, as increasing surfactant concentration may increase the ET due to the high viscosity of the surfactant, which can obstruct the mixing process, leading to an extension of the time required to achieve a homogenous emulsion [24]. In this study, all formulations with varying surfactant concentrations had short ET (less than 1 min), as shown in Table 2. The different concentrations of the surfactant did not influence ET.

### 3.4. Particle Size and PDI

The droplet PS of the emulsion has a significant impact on the rate and amount of drug that is dissolved and absorbed in the GIT after oral administration. A small PS increases the interfacial surface area, leading to enhanced absorption [49]. The SEDDS formulations with the smallest PS and PDI were more promising; therefore, they were selected for CBD loading (Table 2).

### 3.5. Drug Loading

**Figure 3:**
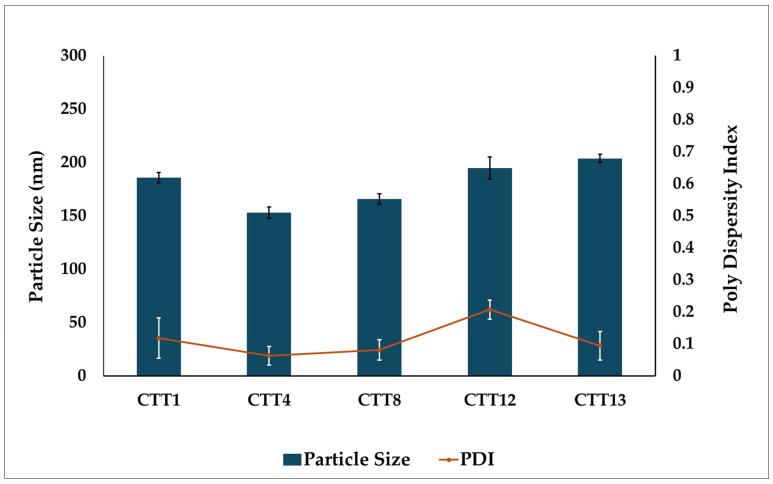
The mean of the particle size and PDI for CBD SEDDS formulations (*n* = 3).

SEDDS formulations (CTT1, CTT4, CTT8, CTT12, and CTT13) with the smallest droplet size (less than 100 nm) and an acceptable standard deviation, as well as a narrow size distribution (PDI below 0.3), were identified as optimal systems and were selected for CBD loading. After loading CBD into the SEDDS formulations, all CBD SEDDS formulations showed a drug content between 100.1 to 105.8%. Their ET was measured and found to remain unchanged, suggesting that adding the drug did not affect the system’s ability to emulsify. However, there was a significant increase in PS. As demonstrated in Figure 3, the average PS of the five CBD SEDDS formulations was approximately 200 nm, which is still considered acceptable for nano-emulsion.

### 3.6. Kinetic Stability

A kinetically stable formulation should withstand a range of stress conditions without exhibiting phase separation and should retain the ability to emulsify spontaneously upon dilution. All CBD SEDDS formulations remained stable after the freeze–thaw and centrifugation tests, showing no phase separation. The emulsification behavior also stayed consistent, as the ET, PS, and PDI showed no change before and after the thermodynamic testing. All formulations exhibited emulsification times of less than 1 min. PS were 197.8 ± 3.7, 154.8 ± 4.0, 160.8 ± 2.0, 199.7 ± 5.5, and 203.9 ± 3.0 nm, with corresponding PDI of 0.19 ± 0.03, 0.11 ± 0.01, 0.09 ± 0.02, 0.27 ± 0.07, and 0.15 ± 0.03 for CTT1, CTT4, CTT8, CTT12, and CTT13, respectively.

### 3.7. In Vitro Drug Release

**Figure 4:**
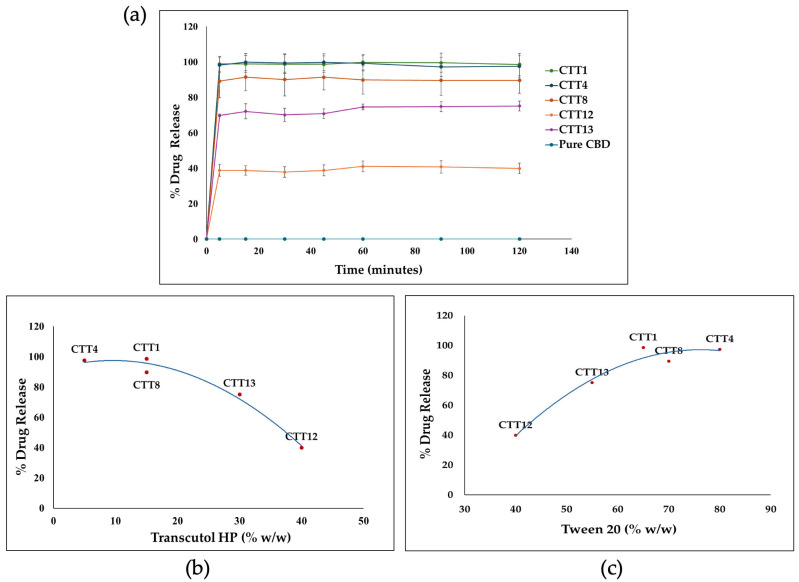
The release profiles of CBD-SEDDS formulations in phosphate buffer pH (6.8). (**a**) The release of pure CBD and CBD SEDDS formulations for two hours, (**b**) the relationship between the release of CBD-SEDDS formulations after two hours and the concentration of cosolvent (Transcutol^®^ HP), and (**c**) the relationship between the release of CBD SEDDS formulations after two hours and the concentration of the surfactant (Tween^®^ 20).

Five SEDDS formulations (CTT1, CTT4, CTT8, CTT12, and CTT13) that passed the previous evaluation were used for the in vitro release test. By directly adding the formulation to the release media, all the CB D formulations showed a remarkable enhancement in CBD release compared to the pure CBD, which was below the detection limit. The release of all formulations reached the maximum drug release within five minutes and maintained the drug solubility without precipitation for up to 120 min. The amount of cosolvent (Transcutol^®^ HP) played a significant role in the CBD release profile. Increasing the amount of Transcutol^®^ HP above 15% *w*/*w* would reduce CBD release. As Transcutol^®^ HP is a cosolvent that may lose its solvent capacity after dilution, it may lead to drug precipitation [50]. As shown in Figure 4, the CBD release profile is inversely proportional to the amount of Transcutol^®^ HP; in contrast, the amount of Tween^®^ 20 is directly proportional to the CBD release. Overall, the formulations containing 15% *w*/*w* or less Transcutol^®^ HP and 65% *w*/*w* or higher Tween^®^ 20 showed drug release of more than 90% (Figure 4).

### 3.8. In Vitro Digestion

**Figure 5:**
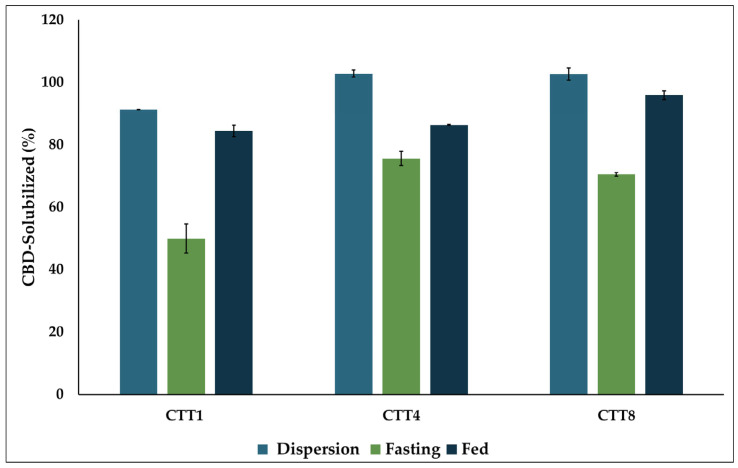
The percentage of CBD solubility after dispersion in the digestive media without pancreatin enzymes and after 30 min of adding the pancreatin enzymes in FaSSIF and FeSSIF.

Lipid-based formulations tend to undergo digestion in the presence of endogenous digestive materials (bile salts, phospholipids, and lipolytic enzymes), which can induce changes in lipid composition and result in the formation of a range of colloidal phases (micelles, vesicles, and liquid crystalline phases) in the intestinal lumen. This change in lipid composition induced by digestion is associated with formulation performance, including the drug’s solubilization capacity and, consequently, its absorption [51,52]. The CBD SEDDS formulations, CTT1, CTT4, and CTT8, showed the highest drug release and were selected for evaluation of their performance in digestive media using an in vitro digestion experiment. After 10 min of formulation dispersion in the digestive media, all formulations dispersed effectively and maintained the drug solubility by at least 90%. The digestion process was initiated upon the addition of lipolytic enzymes and continued for 30 min, as per the literature, which suggests that 30 min is a suitable time for the complete hydrolysis of medium-chain triglycerides (MCT) [53]. Under FaSSIF, all formulations were affected by digestion, but with different degrees, resulting in partial drug precipitation. The formulation composition played a significant role in its digestive sustainability. The CTT1 formulation with a higher oil content (20% *w*/*w*) was more digestible; however, it retained around 50% of its CBD solubility after digestion. In contrast, the other two formulations, CTT4 and CTT8, containing 15% *w*/*w* oil, retained more than 70% of CBD solubility after digestion. On the other hand, in FeSSIF, the digestive media contains higher levels of endogenous emulsifiers, such as bile salts and lecithin, which would help enhance drug solubility after digestion compared to FaSSIF. Furthermore, the formulation’s composition did not affect their performance in FeSSIF (Figure 5). And in the fed state, all three formulations maintained drug solubility of at least 80%.

The increase in the drug solubility in FeSSIF compared to FaSSIF may influence the drug’s susceptibility to the food effect, as the drug’s in vivo bioavailability may increase in the presence of food. In this study, the differences in CBD solubility among the three formulations between the fed and fasted states ranged from 10% to 30%, suggesting that these formulations may help mitigate the food effect on CBD solubility and overall performance.

### 3.9. In Vivo Bioavailability Data

**Figure 6:**
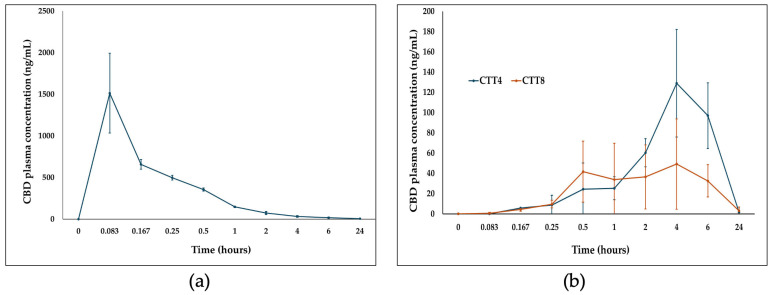
CBD plasma concentration (ng/mL) over 24 h (**a**) after CBD intravenous administration and (**b**) after oral administration of CTT4 and CTT8 formulation.

Formulations CTT4 and CTT8 exhibited the smallest differences in CBD solubility between FaSSIF and FeSSIF and were therefore selected for in vivo pharmacokinetic evaluation in three groups of Sprague–Dawley rats. Two groups received oral doses of 5 mg CBD (equivalent to 20 mg/kg), while a third group received 1 mg CBD intravenously to determine absolute bioavailability. Post-administration, all animals were in good health, with no signs of toxicity. Following IV administration, CBD displayed a rapid onset (T_max_ = 5 min) with an AUC_0–24h_ of 912.79 ng·h/mL, and approximately 70% of total exposure occurred within 4 h (Figure 6a, Table 3). After oral administration, both SEDDS formulations (CTT4 and CTT8) reached peak plasma concentrations at 4 h (T_max_). CTT4 achieved a 2.6-fold higher Cmax (129.03 ng/mL) compared to CTT8 (49.22 ng/mL) (Figure 6b).

CTT8 exhibited a faster initial release, reaching 41 ng/mL within 30 min versus 24.47 ng/mL for CTT4. At 4 h, the AUC_0–24h_ for CTT8 represented 26% of total exposure, while CTT4 accounted for 18%. Overall, CTT4 demonstrated higher AUC_0–24h_ and total AUC_0–24h_ values (1363.40 vs. 562.45 ng·h/mL, respectively). The absolute oral bioavailability of CTT4 and CTT8 was 29.87% and 12.32%, respectively—substantially greater than the reported 6% fasted-state bioavailability of the marketed formulation Epidiolex^®^ [20].

CTT4 and CTT8 showed highly similar results in their in vitro evaluations, as, for particle size, CTT4 showed 152.8 ± 5.35 nm and 165.8 ± 4.99 nm for CTT8. Furthermore, both formulations showed more than 90% of drug release. In addition, for in vitro digestion in the fasting state, CTT4 maintained 75.6 ± 2.29% of CBD solubility, while CTT8 maintained 70.49 ± 0.60%. In contrast, the in vivo results showed a significant difference between the two formulations. CTT4 showed a C_max_ 2.6-fold higher than CTT8 and an absolute bioavailability 2.4-fold higher than CTT8. The main explanation for these results is the formulation composition, as CTT4 contained a higher amount of the inhibitory non-ionic surfactant Tween^®^ 20 (80% *w*/*w* compared to 70% *w*/*w* in CTT8). Increasing the amount of Tween^®^ 20 may enhance liver enzyme inhibition, thereby decreasing CBD metabolism and increasing its bioavailability.

## 4. Conclusions

In this research study, a self-emulsifying drug delivery system (SEDDS) was developed to enhance cannabidiol’s (CBD) solubility, dissolution rate, and oral bioavailability. Among the prepared formulations, CTT4 and CTT8 showed complete in vitro drug release, rapid emulsification time, nano-sized particles, and prolonged solubilization during in vitro digestion in both fed and fasted states, suggesting their potential to reduce food intake variability. The in vivo pharmacokinetic study demonstrated that CTT4 produced notably greater systemic exposure, resulting in a 2.4-fold increase in absolute oral bioavailability compared to CTT8. This improvement is likely due to the higher proportion of the non-ionic surfactant Tween^®^ 20 in the CTT4 formulation, which may help limit first-pass metabolism and facilitate more efficient absorption of CBD. In conclusion, these findings highlight the importance of formulation composition, particularly surfactant type and concentration, in optimizing CBD bioavailability. Moreover, SEDDS represents a promising approach in the development of improved oral CBD formulations with reduced dosing variability and potentially enhanced therapeutic outcomes.

## Figures and Tables

**Table 1:** The compositions of the 16 SEDDS placebo formulations.

Formulation	Capryol^®^ 90 (% *w*/*w*)	Tween^®^ 20 (% *w*/*w*)	Transcutol^®^ HP (% *w*/*w*)
CTT1	20	65	15
CTT2	80	10	10
CTT3	10	80	10
CTT4	15	80	5
CTT5	50	40	10
CTT6	25	70	5
CTT7	30	60	10
CTT8	15	70	15
CTT9	40	45	15
CTT10	60	25	15
CTT11	10	10	80
CTT12	20	40	40
CTT13	15	55	30
CTT14	25	15	60
CTT15	40	15	45
CTT16	50	20	30

**Table 2:** SEDDS formulation ET, PS, and PDI of SEDDS (mean ± S.D., *n* = 3).

Formulation	E T (sec.)	P S * (nm)	PDI
CTT1	57	13.85 ± 1.11	0.24 ± 0.02
CTT2	_	_	_
CTT3	20	17.76 ± 14.12	0.20 ± 0.08
CTT4	15	11.16 ± 2.16	0.18 ± 0.47
CTT5	10	266 ± 17.36	0.37 ± 0.02
CTT6	13	82.66 ± 2.36	0.54 ± 0.04
CTT7	10	89.57 ± 53.77	0.85 ± 0.24
CTT8	11	10.49 ± 0.57	0.18 ± 0.04
CTT9	5	225.00 ± 50.11	0.46 ± 0.16
CTT10	-	-	-
CTT11	5	405.50 ± 119	0.39 ± 0.09
CTT12	6	63.59 ± 1.17	0.20 ± 0.02
CTT13	25	11.50 ± 0.87	0.17 ± 0.06
CTT14	5	146.50 ± 49.82	0.38 ± 0.09
CTT15	_	_	_
CTT16	_	_	_

* Particle size is reported as Z-average.

**Table 3:** The pharmacokinetic parameters following CBD intravenous administration and after CBD SEDDS (CTT4 and CTT8) oral administration. All values represent the mean ± standard deviation.

Group	C_max_ (ng/mL)	T_max_ (h)	AUC_0–4h_ (ng⋅h/mL)	AUC_0–24h_ (ng⋅h/mL)
CBD-IV	1513.63 ± 480.42	0.083	649.80 ± 65.45	912.79 ± 159.86
CTT4	129.03 ± 53.15	4	249.33 ± 29.05	1363.16 ± 245.13
CTT8	49.22 ± 44.55	4	147.01 ± 94.46	549.12 ± 199.94

## Data Availability

The authors declare that all data will be available upon request.

## Abbreviations

The following abbreviations are used in this manuscript:

CBD	Cannabidiol
THC	Tetrahydrocannabinol
CB	Cannabinoid receptor
ECS	Endocannabinoid system
FAAH	Fatty acid amide hydrolase
LBDDS	Lipid based drug delivery system
GIT	Gastrointestinal tract
P-gp	P-glycoprotein
SEDDS	Self-emulsifying drug delivery system
PS	Particle size
PDI	Polydispersity index
ET	Emulsification time
PVDF	Polyvinylidene fluoride
IACUC	Institutional animal care and use committee
UPLA	Ultra-performance liquid chromatography
MRM	Multiple reaction monitoring
LLOQ	Lowest limit of quantification
AUC	Area under the curve
MTC	Medium-chain triglyceride
HLB	Hydrophilic lipophilic balance
